# Structural Health Monitoring: Advanced Sensing, Diagnostics and Prognostics

**DOI:** 10.3390/s25051313

**Published:** 2025-02-21

**Authors:** Bing Li, Yongbo Li, Khandaker Noman

**Affiliations:** 1 School of Aeronautics, Northwestern Polytechnical University, Xi’an 710072, China; bingli@nwpu.edu.cn; 2 Aircraft Strength Research Institute of China, Xi’an 710065, China; 3 School of Civil Aviation, Northwestern Polytechnical University, Xi’an 710072, China

Structural Heath Monitoring (SHM) can be considered one of the most prominent emerging components of modern engineering applications. Ensuring the reliability and safety of machinery and infrastructure has become more challenging due to their increasing complexity. Over the course of time, SHM has evolved significantly beyond its conventional foundations in aeronautical, civil, and mechanical engineering, with the discovery of applications in domains such as nuclear energy, maritime constructions, and wind turbine technology. The ability to detect failures earlier and forecast the remaining usable life (RUL) of structures has become crucial over the years. Early identification not only prevents catastrophic failures but also enhances maintenance strategies, saving both money and time. In recent years, different advanced sensing technologies, intelligent data-driven strategies, and innovative diagnostic and prognostic methodologies have witnessed revolutionary advancement. The integration of different analytical methods like non-destructive testing (NDT), artificial intelligence-based detection, vibration, and wave analysis has improved the precision and efficiency of conditioning monitoring. Advancements in these areas provide substantial insights into structural behavior, improving dependability, optimizing performance, and reducing maintenance costs. This Special Issue of *Sensors* aims to gather recent research findings and present the latest advancements in Structural Health Monitoring (SHM) in relation to advanced sensing, diagnostics, and prognostics. Overall, 13 different research contributions are featured in this collection, presenting groundbreaking applications of advanced sensing, diagnostics, and prognostics in the field of Structural Health Monitoring (SHM).

To enhance the finite element model updating (FEMU) process for aging highway viaducts, Hekič et al. used both acceleration- and strain-based assessments on a multi-span concrete viaduct over 50 years old (Contribution 1). In this research, the authors used mid-span strain readings from large trucks for strain-based FEMU and frequencies/mode shapes for acceleration-based FEMU. Optimization methods, such as residual reduction and error-domain model falsification (EDMF), were used to enhance structural parameters. The findings demonstrate that the integration of strain data improves the accuracy of FEMU, showing an estimated 20% increase in the viaduct’s design stiffness and a 25–50% overestimation of internal girder stiffness. The advantages of EDMF in producing physically significant updates in bridge model calibration are highlighted.

Deriving coupled ordinary differential equations for the first two modes in in-plane, out-of-plane, and torsional directions, Cui et al. investigated the spatial galloping behavior of iced conductors under multimodal coupling (Contribution 2). This study analyzed critical conditions within the wind speed–sag parameter space and classified galloping patterns into five distinct regions. The results obtained indicate that single-mode galloping exhibits elliptical motion, whereas coupled-mode galloping follows an “8”-shaped trajectory. The theoretical understanding of multimodal galloping in transmission lines, which provides a basis for designing anti-galloping measures, was enhanced by this study.

Hong et al. proposed a tensor optimization-based robust interval prediction method in order to forecast intermittent demand for spare parts (Contribution 3). The authors performed the integration of tensor decomposition with a stacked autoencoder to smooth abnormal demand variations while preserving intrinsic evolutionary trends. To enhance prediction reliability, an adaptive prediction interval algorithm was designed using LightGBM estimators and a dynamic update mechanism. Improved forecasting for small-sample intermittent time series and provides a reliable elastic prediction interval. This study offers a robust solution for intelligent inventory management.

Janeliukstis et al. proposed a wavelet-based output-only damage detection method for composite structures, using continuous wavelet transform (CWT) methods to extract modal features such as resonant frequencies and damping ratios (Contribution 4). The extracted features were used to construct a statistical damage detection scheme based on kernel density estimation (KDE), where deviations in modal features were identified via the Euclidean distance between KDE centroids. Experimental validation on glass-fiber-reinforced polymer cylindrical specimens demonstrated that the proposed method achieved comparable accuracy to the Mahalanobis distance metric while providing a simpler and more interpretable damage indicator.

Lu et al. simulated the random traffic flow of heavy vehicles via the incorporation of the R-vine Copula model and an improved Latin hypercube sampling (LHS) method (Contribution 5). Weigh-in-motion data were used in this study to examine correlations in vehicle weight, establishing an ideal R-vine Copula model for describing the relationships among vehicle weight characteristics. An improved LHS method was introduced for enhancing sampling accuracy to ensure a more authentic distribution of traffic flow characteristics. Finally, as visible from the load effect analysis, the consideration of vehicle weight correlations yields more conservative and realistic structural safety and assessments compared to the traditional Monte Carlo methods.

Estimating the torsional stiffness using an adaptive extended Kalman filter (AEKF) with a forgetting factor update, Park et al. monitored crack development in rotating shafts. To implement the AEKF, a dynamic system model was developed that allowed the real-time detection of torsional stiffness reduction due to cracks (Contribution 6). Results from the simulation and experiment demonstrated that the method successfully tracked the stiffness changes. Also, the method quantitatively evaluated the fatigue crack growth. This approach relies on the cost-effective rotational speed sensors, which makes it a viable solution for the structural health monitoring of rotating machinery.

Single-sensor engine multi-type fault detection, conducted via the integration of a variational mode decomposition (VMD) method with a Random Forest (RF) classifier, was investigated by Tang et al. (Contribution 7). Through decomposition under multiple operating conditions, the spectral energy distribution of engine signals was obtained. They also optimized the mode number and penalty term to enhance the mode separation and decomposition efficiency. The construction of a future set, including unit bandwidth energy, center frequency, and a maximum singular value, was completed and input into RF for classification. The results from comparative experiments showed that the proposed IVMD-RF method outperformed all the other deep learning approaches in both accuracy and training efficiency, demonstrating effectiveness in cross-speed fault diagnosis, with minimal training data and low hardware requirements.

To detect the axially loaded beams subjected to seasonal thermal variations via principal component analysis (PCA), Berardengo et al. developed a short-training damage detection method (Contribution 8). In this particular approach, PCA is applied to vibration-based damage features to filter out temperature effects and improve detection reliability, even given the constraints of a limited training set. Both numerical simulations and experimental studies on a tie-rod structure, demonstrating superior robustness compared to the conventional Mahalanobis squared distance (MSD)-based approach, validated this method. The results obtained indicate that the proposed PCA-based strategy effectively isolates the damage-sensitive components while suppressing environmental variations, making it a suitable solution for structural health monitoring under varying thermal conditions.

Zhuang et al. used an IBA-ISMO-based method integrating variational mode decomposition (VMD) and sample entropy and diagnosed the rolling bearing faults (Contribution 9). VMD algorithms were applied to decompose vibration signals into intrinsic mode components (IMFs). The sample entropy was extracted as a feature for fault identification. During the optimization of the sequence minimization optimization (ISMO) classifier’s parameters, enhanced classification accuracy was ensured via an improved bat algorithm (IBA). Experimental verification using the CWRA dataset showed that the proposed IBA-ISMO model outperformed conventional methods in fault recognition, demonstrating robustness in detecting different bearing fault types under variable working conditions.

Park et al. extracted degradation features for the prognostics of an extruder screw using multi-source monitoring data from a real micro-extrusion system (Contribution 10). The operational data were utilized in this work to develop a prognostic method for predicting screw wear, addressing the challenge of obtaining real-world run-to-failure data. Based on physical and mechanical properties, integrating motor load, head pressure, and puller speed to estimate screw deterioration, degradation features were derived. It is visible from experimental validation that the extracted feature exhibited monotonic degradation behavior, enabling the accurate prediction of remaining useful life. A practical solution for monitoring extrusion systems health in industrial applications is also provided by the proposed method.

In Jia et al.’s work (Contribution 11), high-precision features were extracted for aircraft attitude sensor fault diagnosis using a RepVGG-based convolutional neural network with an SENet attention mechanism. In this particular method, the transformation of time domain sensor signals was performed to yield time–frequency representations, and SENet was used to allocate weights for both signal domains. Following that, the weighted features were fed into RepVGG for deep feature extraction and classification. Experimental observation validates the achievement of the proposed model as an optimal balance between diagnostic accuracy and computational efficiency, making it suitable for real-time aircraft fault diagnosis.

A TCP acceleration algorithm was developed by Liu et al. (Contribution 12) for aerospace–ground service networks. This method leverages historical transmission characteristics and congestion control optimizations. This study introduced BoostTCP, a learning-based algorithm that dynamically adjusts transmission rates based on end-to-end delay variations, feedback packet intervals, and random packet loss factors, and addressed the inefficiencies of standard TCP in high-bandwidth, long-delay networks. The comparative evaluations with conventional TCP congestion control algorithms demonstrated that BoostTCP significantly improved throughput, fairness, and bandwidth utilization in both simulated and real-world aerospace networks, and the results suggest that BoostTCP provides a promising solution for high-speed satellite data transmission.

Yang et al. (Contribution 13) reviewed the status and applications of hydraulic pump fault diagnosis, summarizing existing methodologies in fault detection, prediction, and health management. The classification of hydraulic pump fault diagnosis methods into signal processing-based approaches, artificial intelligence-driven techniques, and mechanism analysis-based methods was performed in this paper. This work also addressed several key challenges such as sensor selection, model construction, and multi-source data fusion, and the growing role of AI in improving fault recognition accuracy was highlighted by the comparative analysis of reviewed methods. Insights into future trends, emphasizing the need for hybrid techniques that integrate physical modeling with data-driven strategies for enhanced fault diagnostics and prognostics, were also provided.

The editors express their sincere gratitude to all the contributing authors for the outstanding research contributions. Special thanks are also due to the reviewers for their valuable feedback, which significantly helped to enhance the overall quality of this Special Issue. Lastly, we also appreciate the support of the editorial board of *Sensors* for facilitating the dissemination of these important studies.

